# *Pandoraea* Infections in Humans—A Systematic Review

**DOI:** 10.3390/jcm13226905

**Published:** 2024-11-16

**Authors:** Afroditi Ziogou, Alexios Giannakodimos, Ilias Giannakodimos, Andreas G. Tsantes, Petros Ioannou

**Affiliations:** 1 Department of Medical Oncology, Metaxa Cancer Hospital of Piraeus, 18537 Piraeus, Greece; 2 Department of Urology, Attikon University General Hospital of Athens, 12462 Athens, Greece; 3 Laboratory of Hematology and Blood Bank Unit, School of Medicine, Attikon University General Hospital of Athens, National and Kapodistrian University of Athens, 12462 Athens, Greece; 4 School of Medicine, University of Crete, 71003 Heraklion, Greece

**Keywords:** *Pandoraea*, infection, pneumonia, bacteremia, endocarditis, osteomyelitis

## Abstract

**Background/Objectives:** *Pandoraea* species are Gram-negative, aerobic, rod-shaped bacteria that belong to the Burkholderiaceae family and the Betaproteobacteria class. Despite their rare occurrence in the general population, they have been increasingly observed as the causes of infection in immunocompromised individuals or patients with severe comorbidities. The present review seeks to examine all documented cases of *Pandoraea* spp. infections in humans, focusing on data related to epidemiology, microbiology, antimicrobial susceptibility, treatment options, and mortality rates. **Methods:** A systematic review was conducted through a literature search of the PubMed/MedLine and Scopus databases. This review is subjected to certain limitations regarding the data accuracy or pathogen identification molecular techniques applied in the studies. **Results:** In total, 29 studies provided information on 43 patients with *Pandoraea* spp. infections. The mean age of the patients was 42 years, and 58% were male. Cystic fibrosis was these patients’ most prevalent risk factor (39.5%). The most frequently reported types of infection were lower respiratory tract infections (74.41%) and bacteremia (30.23%), followed by infective endocarditis, pancreatitis, upper respiratory tract infection, and osteomyelitis (4.65%). *P. apista* was the most regularly isolated species (37.2%), while antimicrobial resistance was lower for carbapenems, especially for imipenem (17.14%). The most commonly administered antibiotics included carbapenems (82%), cephalosporins, and trimethoprim/sulfamethoxazole (35.89%). The infection outcome primarily depended on the type of infection; mortality rates were high (30.23%) and particularly elevated for bloodstream infections. The protocol for this review was registered in Prospero (ID: CRD42024579385). **Conclusions:** Due to *Pandoraea*’s unique antimicrobial resistance pattern and capacity to induce severe infection, clinicians should include it when making a differential diagnosis, especially in patients with severe comorbidities and immunodeficiency.

## 1. Introduction

The discovery of new microorganisms has become increasingly common, resulting from genetic techniques like 16S rRNA gene sequencing [1]. These advanced methods offer greater accuracy in diagnosing infections caused by microorganisms that are difficult to detect using traditional microbiological approaches [2].

*Pandoraea* genus is an emerging Gram-negative, obligate aerobic, rod-shaped bacterium that belongs to the Burkholderiaceae family. This pathogen was initially described in 2000 to accommodate bacteria from Pseudomonas rRNA homology group II [3]. At present, eleven *Pandoraea* species have been described [4,5]. These pathogens may be isolated from various environmental sources and biological specimens, including respiratory tract, urine, or blood samples [6]. Although *Pandoraea* spp. are considered opportunistic pathogens, numerous cases of severe infection have been described, especially in patients with severe underlying conditions. This is attributed to the pathogens’ capacity to induce a pronounced pro-inflammatory response [7,8]. Most infections involve the respiratory tract and affect cystic fibrosis (CF) patients [9]. However, cases of *Pandoraea* bacteremia and even endocarditis or osteomyelitis have been reported in the literature [10,11]. Due to the challenges encountered in the pathogen’s detection and the phenotypic similarity with *Ralstonia* or *Burkholderia* species, these genera are often misidentified, leading to the underdiagnosis of *Pandoraea* infections globally [10]. Accurate identification necessitates advanced microbiological techniques, such as 16s rRNA gene sequencing [12]. Until today, there have been no officially established antibiotic susceptibility breakpoints for *Pandoraea* spp.; these pathogens are considered multi-drug-resistant, and most present high resistance to β lactams and aminoglycosides. Identifying these peculiar resistance patterns is essential for immediate infection control [3]. There are no established treatment guidelines for *Pandoraea* infections; most cases are empirically treated while the results of antimicrobial susceptibility testing are pending. The difficulty in establishing treatment guidelines and applying empirical treatment may derive from the limited number of published studies globally. The mortality rates for these infections are relatively high, with outcomes largely influenced by the infection site and the patient’s underlying health conditions.

The main objective of this study was to review all published cases of *Pandoraea* spp. infections in humans, focusing on epidemiology and mortality data. Additionally, it aims to describe the microbiology, antimicrobial susceptibility, and treatment of these infections, address existing areas of uncertainty, including risk factors and treatment options, and expand the limited information available in the literature on this emerging microorganism.

## 2. Materials and Methods

### 2.1. Search Strategy and Inclusion and Exclusion Criteria

The present systematic review was performed according to the Preferred Reporting Items for Systematic Reviews (PRISMA) and Meta-Analyses guidelines [13]. The protocol was registered in Prospero (ID: CRD42024579385). Information on *Pandoraea* species-related human infections was extracted and gathered from the existing literature. The principal objective was to analyze these infections’ mortality rates and epidemiology data. Additionally, the secondary goals included demonstrating information on the specific infection sites, providing thorough clinical profiles of all affected patients, microbiological data, and treatment regimens administered to individuals affected with *Pandoraea* spp. infections. For this review, two investigators (A.Z. and A.G.) independently searched PubMed/Medline and Scopus databases for potential articles reporting on all *Pandoraea* spp. infections until 25 September 2024, using a pre-defined template. The following keywords were used for the search strategy: “*Pandoraea*” AND (“infection” OR “bacteremia” OR “endocarditis” OR “peritonitis” OR “pneumonia” OR “osteomyelitis”). Any occurring dispute was resolved by a senior investigator’s (P.I.) intervention. The inclusion criteria for the present review consisted of studies presenting original data, such as case series, case reports, and cohort studies that provided information on the epidemiology and clinical outcomes of *Pandoraea* spp. infections in humans. Only studies published in English were included to optimally comprehend all clinical details. Reviews and systematic reviews with aggregated data were not considered. Animal studies and articles that lacked full-text access or did not provide sufficient data on patient mortality and epidemiology were also excluded. To ensure comprehensive coverage, the references of all included articles were reviewed to detect any studies that may have been missed in the initial search. 

### 2.2. Data Extraction and Definitions

The following data were collected from each included article: publication year, type of article, country of origin, patients’ demographics (age, gender), patients’ pertinent medical history, data on infection, and relevant microbiological features, such as exact infection site, complications, and identified pathogen and antibiotic resistance, as well as treatment options and clinical outcome (survival or mortality). The correlation between mortality and the initial infection was documented as per the authors of each study. The exclusion of studies in languages other than English may potentially lead to a sample bias; however, the number of these articles was small. 

### 2.3. Statistical Analysis

Descriptive statistics were applied for numerical variables, presenting mean values with their corresponding standard deviations (SDs) or medians with interquartile ranges (25–75%) in a skewed data distribution. Categorical variables were exhibited by frequencies and percentages to provide a detailed overview. Patients included in the case series were considered individual case reports to facilitate the evaluation of variables of interest.

A univariate linear regression analysis was conducted to identify factors associated with patients’ overall mortality. Given the small number of values provided for most patients’ characteristics, the small clinical relevance of some of them, and the small number of patients included in the present analysis, only gender, age, history of cystic fibrosis, previous use of antimicrobials, bloodstream infection, fever, and sepsis were included in the univariate linear analysis. Statistics were calculated with GraphPad Prism 6.0 (GraphPad Software, Inc., San Diego, CA, USA). A multivariate logistic regression analysis evaluated the effect of factors previously identified in the univariate analysis model associated with all-cause mortality with a *p* < 0.1. Multivariate analysis was performed using SPSS version 23.0 (IBM Corp., Armonk, NY, USA).

## 3. Results

### 3.1. Included Studies’ Characteristics

**Figure 1:**
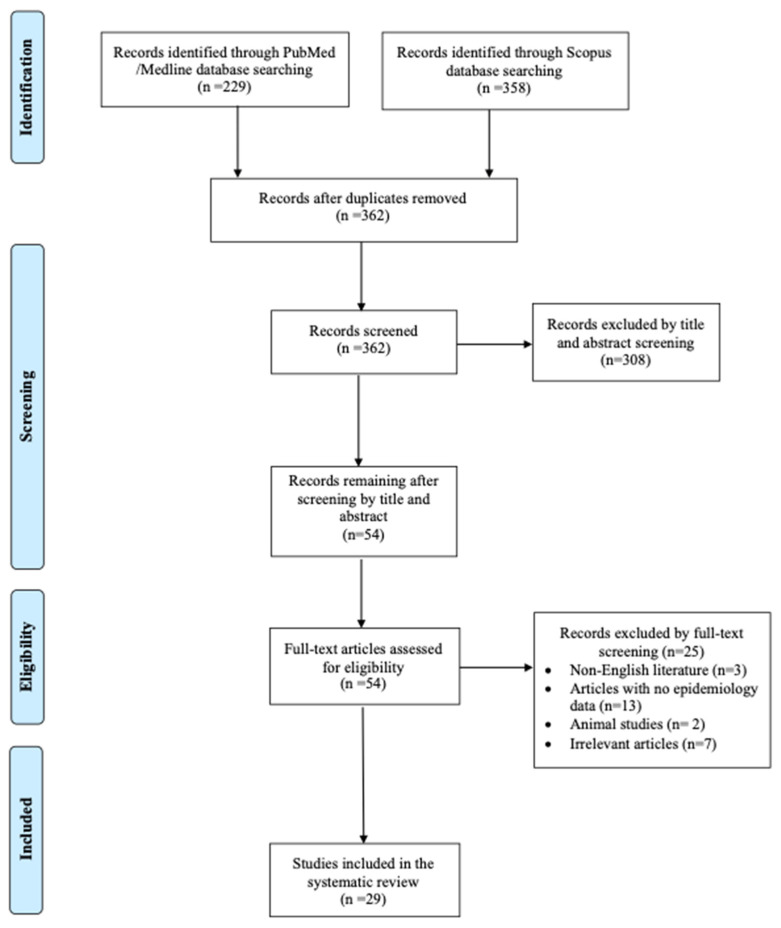
Flow diagram of study inclusion.

**Figure 2:**
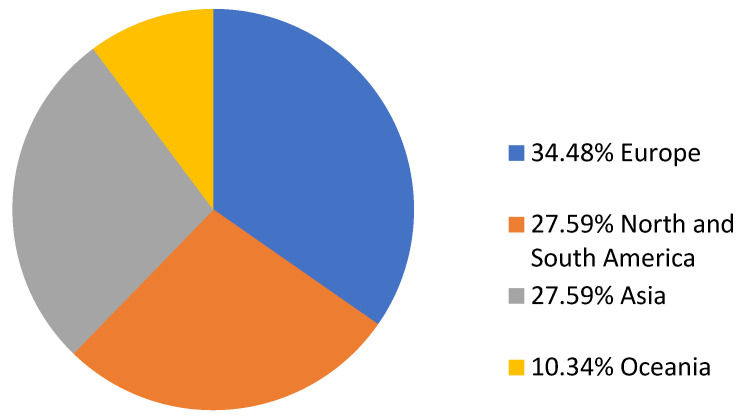
Pie chart of geographical distribution of *Pandoraea* spp. infections worldwide.

The literature search of the PubMed and Scopus databases retrieved 362 non-duplicate studies. After record screening and a snowball procedure, 29 articles met the inclusion criteria and were included in the analysis. These 29 studies provided data on 43 patients. A flow diagram of the selection process is depicted in Figure 1. Among them, ten studies were conducted in Europe, eight in Asia, eight in North and South America, and three in Oceania. There were 26 case reports, 2 case series, and 1 letter to the editor [6,8,9,10,11,12,14,15,16,17,18,19,20,21,22,23,24,25,26,27,28,29,30,31,32,33,34,35]. Table S1 shows the main characteristics of the studies. Figure 2 shows the geographical distribution of the cases.

### 3.2. Epidemiology of Pandoraea spp. Infections in General

The mean age of the patients with *Pandoraea* infection was 42.08 years, ranging from 1.5 to 79 years, while 58.14% (25 out of 43) were male. In regard to the patients’ medical history, 17 patients (39.5%) were diagnosed with cystic fibrosis, and 15 patients (34.88%) were administered antibiotics in the previous three months. Additionally, eight patients (18.6%) were immunosuppressed, seven individuals (16.27%) had a history of active malignancy, and among them, five patients (11.62%) were diagnosed with hematologic malignancies. Of note, recent organ transplantation and the presence of a central venous catheter (CVC) were documented in five individuals (11.62%), while severe trauma was noted in four patients (9.3%). The characteristics of the patients with *Pandoraea* species infection are presented in Table 1. 

### 3.3. Microbiology and Antimicrobial Resistance of Pandoraea Infections in General

*Pandoraea* spp. were isolated from tracheobronchial secretion or sputum cultures in 29 patients (67.44%), blood cultures in 9 patients (20.93%), and CVC cultures in 1 patient (2.32%); of note, in 1 patient (2.32%) the pathogen was detected in a lung biopsy, in another patient (2.32%), in the peritoneal fluid and, in 1 patient (2.32%), in bone culture. *Pandoraea apista* was the most commonly identified species (37.2%); other detected species included *Pandoraea commovens* in 10 patients (23.25%), *Pandoraea sputorum* in 8 patients (18.6%), *Pandoraea pnomenusa* in 6 patients (13.95%), and *Pandoraea pulmonicola* in 3 patients (6.97%). A few rare species, including *Pandoraea nosoerga*, *Pandoraea vervacti*, and *Pandoraea oxalalivorans,* were also identified. The infection was polymicrobial in 23 individuals (53.48%); the most frequently co-isolated pathogen was *Pseudomonas aeruginosa* in 16 patients (37.2%), while *Candida* was present in 6 patients (13.95%). Of note, one patient (2.32%) was found positive for SARS-CoV-19. The identification of the pathogen was achieved through genetic testing, particularly using 16s-rRNA sequencing in 11 individuals (25.58%) and matrix-assisted laser desorption/ionization time-of-flight mass spectrometry (MALDI-TOF MS) in 24 patients (55.81%). 

Concerning antimicrobial resistance, disk diffusion was the most common method applied in 18 patients (41.86%). Antimicrobial resistance was high for Penicillin, Aztreonam, Macrolides, Chloramphenicol, Aminoglycosides, Piperacillin, Quinolones, and Cephalosporins. The antimicrobial resistance rates are depicted in Table 2. 

### 3.4. Clinical Presentation of Pandoraea Infections 

The most frequently observed *Pandoraea* infection site was the lower respiratory system in 32 patients (74.41%), followed by bloodstream infection in 13 patients (30.23%). Upper respiratory infection, osteomyelitis, pancreatitis, and endocarditis were noted in two patients (4.65%), while infections of the liver and skin were reported in one patient, respectively (2.32%). Based on the available data, the duration of symptoms varied between 0 days (acute onset of infection) and 180 days. Interestingly, 42.85% (12 out of 28 patients) presented symptoms of the infections intermittently for several years, ranging from 1.5 to 11 years.

### 3.5. Treatment and Outcomes of Pandoraea Infections 

Concerning antimicrobial treatment, 69.23% of the included patients received antibiotics (27 out of 39 with the available data). The most frequently administered antibiotics were carbapenems at 82.05% (32 out of 39 patients) and cephalosporins and Trimethoprim/sulfamethoxazole at 35.89% (14 patients). Additionally, aminoglycosides were used in 28.2% (11 patients), quinolones in 25.64% (10 patients), macrolides in 23.07% (9 patients), and tetracyclines in 20.51% (8 patients). Other less common antibiotics used included rifampicin and piperacillin/tazobactam in 17.94% (seven patients), colistin and tigecycline in 12.82% (five patients), vancomycin in 10.25% (four patients), aminopenicillins in 5.12% (two patients), sulbactam, aztreonam, daptomycin, linezolid, and hydroxychloroquine in 2.56% (one patient). Notably, only 2.43% (1 out of 41 patients based on the available data) did not receive antibiotics. Operative interventions were conducted along with antimicrobial treatment in 16.6% (7 out of 42 patients), while 7.14% (3 out of 42 patients) underwent surgical procedures without antibiotic administration. The median duration of treatment for the survivors was 13 days. The overall mortality rate was 30.23% (13 out of 43 patients), while the mortality rate attributed specifically to *Pandoraea* infection was 20.93% (9 patients). The total characteristics of the different types of *Pandoraea* species infections are depicted in Table 3. 

### 3.6. Respiratory Infection Due to Pandoraea

Thirty-two patients who developed upper and lower respiratory infections due to *Pandoraea* spp. were identified. The mean age of these patients was 38.07 years, ranging from 1.5 to 75 years, and 53.12% (17 out of 32 patients) were male. Among these patients, 53.12% (17 out of 32) had been diagnosed with cystic fibrosis, 37.5% (12 patients) had been administered antibiotics in the previous three months, 18.75% (6 patients) were immunosuppressed, and 12.5% (4 patients) presented with active malignancy; in 75% (three out of four) of this patient group, the malignancy was hematologic and only one patient (25%) was on chemotherapy. Of note, 12.5% (four patients) had been subjected to organ transplantation; lung transplantation was conducted on three of these individuals. Finally, neutropenia and a CVC were present in 6.25% (two patients). In most patients (62.5%), the infection was polymicrobial; Pseudomonas aeruginosa was the most frequently isolated pathogen (50%). Respiratory failure was observed in 87.5% (28 patients). Sepsis and septic shock were reported in 31.25% (eight patients) and 15.62% (five patients), respectively. Heart failure and need for hemodialysis were noted in 9.37% (three patients). The mean duration of treatment for patients with respiratory infections due to *Pandoraea* was 30.23 days. Antibiotics were used in the majority of patients (93.75%), surgical interventions were conducted in 18.75% (six patients), while in 12.5% (four patients) both antibiotics and surgical procedures were applied. The overall mortality was 31.25% (10 patients), and mortality directly associated with *Pandoraea* infection was 21.87% (7 patients).

### 3.7. Bacteremia Due to Pandoraea

Bacteremia was detected in thirteen patients. The mean age of this patient group was 45.85 years, varying from 16 to 79 years, and 76.92% (10 patients) were males. Previous antibiotic administration during the last three months, immunosuppression, the presence of a CVC line, and organ transplantation were recorded in 30.76% (four patients). Active malignancy and a history of surgery in the last three months were noted in 23.07% (three patients), and a history of cystic fibrosis was noted in 15.38% (two patients), while end-stage renal disease, a history of rheumatic fever, intravenous drug use, and autoimmune syndrome in 7.7% (one patient) were noted. Polymicrobial infection was observed in 23.07% (three patients); *Clostridium perfingens*, *Staphylococcus* spp., *Candida* spp., and *Pseudomonas aeruginosa* were isolated along with *Pandoraea* spp. from these individuals. Among all the cases with bacteremia, 76.92% (10 patients) developed fever, 46.15% (6 patients) sepsis, and 30.76% (4 patients) septic shock. Organ failure occurred in 46.15% (six patients); 83.33 (five out of six patients) presented with respiratory failure, while 33.3% (two patients) presented with heart and kidney failure, respectively. Hemodialysis was required for all the patients who presented with kidney insufficiency. Finally, 16.6% (one patient) developed liver insufficiency. The mean duration of treatment was 30.6 days. Antibiotics were applied to 84.61% of patients (11), a surgical approach was used in 30.7% (4), and both methods were used in 23.07% (3). The overall mortality was estimated at 38.46% (five patients), and mortality was caused specifically by *Pandoraea* spp. in 30.76% (four patients).

### 3.8. Infective Endocarditis Due to Pandoraea

In the present systematic review, 4.65% (2 out of 43 patients) developed infective endocarditis caused by *Pandoraea* spp. The mean age was 39.5 years, and 100% of cases were males. All patients (100%) had prosthetic cardiac valves. One patient was an intravenous drug user, had a CVC, underwent cardiac surgery, and received antibiotics within the previous three months. The second patient had a metallic valve and presented rheumatic fever. In both patients (100%), *P. pnomenusa* was the only identified pathogen. The infection was community-acquired in 50% (one patient), while in 50%, it was hospital-acquired. The diagnosis was established by applying Duke’s criteria in both patients (100%) and transesophageal or transthoracic ultrasound (50%, respectively). Fever was present in both cases (100%); however, septic shock, multiple organ failure, and the need for hemodialysis occurred in one patient (50%). None of the patients exhibited immunologic or embolic complications. The mean treatment duration was 28 days. All patients (100%) received antibiotics; one (50%) underwent CVC removal and replacement. The overall mortality was 50% (one patient), and *Pandoraea*-specific mortality was also 50% (one patient). 

### 3.9. Osteomyelitis Due to Pandoraea

Osteomyelitis was recorded in 4.65% (2 out of 43 patients). The mean age was 61 years, and 100% were males. Risk factors were present only in one patient and included immunosuppression due to hematologic malignancy accompanied by neutropenia and recent antibiotic use; lower respiratory infection was also present in this patient. *P. apista* and *P. commovens* were identified in these two patients. In the first patient, osteomyelitis was located in the skull, leading to hearing loss. The second patient presented with mediastinitis complicated with sepsis and respiratory failure. Both patients received antibiotics, specifically quinolones (50%) and carbapenems (50%). Surgical interventions were not performed. The overall mortality was 50% (one patient), although no deaths were attributed to *Pandoraea* spp.

### 3.10. Other Infections Due to Pandoraea

Pancreatic infections were detected 4.65% (2 out of 43 cases). The mean age of the patients was 56 years, and 50% were male (one patient). One patient was recently subjected to ERCP that caused pancreatitis, while the second patient had a medical history of diabetes mellitus-associated chronic kidney disease. *P. commovens* was isolated after an intra-abdominal puncture in both patients. All patients (100%) needed hemodialysis and intensive care, while one (50%) was reported to have respiratory failure. All patients received antibiotics and underwent surgical interventions. The overall mortality was 50%, but no deaths were caused by *Pandoraea* infection. 

Skin infection was reported in 2.32% (1 out of 43 patients). The patient was a 46-year-old woman with severe burns. Bacteremia was also present. Several *Pandoraea* species were identified, such as *P. sputorum*, *P. vervacti*, and *P.oxalalivorans*. The patient developed septic shock and needed intensive care. The survival rate was 100%.

### 3.11. Results of the Statistical Analysis

A univariate linear regression analysis of the overall mortality with gender, age, history of cystic fibrosis, previous use of antimicrobials, bloodstream infection, fever, and sepsis was performed, and fever and sepsis were identified as being positively associated with the overall mortality (*p* = 0.0143 and *p* = 0.0017, respectively). A multivariate logistic regression analysis, after including those two parameters (the only ones with a *p* < 0.1 in the univariate analysis), did not identify any factor to be independently associated with the overall mortality. Table 4 shows the regression analysis of the overall mortality in Listeria IE patients.

## 4. Discussion

The present systematic review highlights the characteristics of infections caused by *Pandoraea* species, drawn from various studies that offer detailed insights into epidemiology, microbiology, clinical presentations, treatment, and clinical outcomes. The most frequently reported infections were respiratory, bacteremia, endocarditis, osteomyelitis, pancreatitis, and skin and liver infections. Among the *Pandoraea* species, *Pandoraea apista* was the most commonly identified. Carbapenems emerged as the most widely utilized antimicrobial agent in treatment. This review also underlines that the overall mortality from *Pandoraea* infections was relatively high.

Due to the limited number of cases reported on *Pandoraea* spp. infections in the current literature, establishing precise epidemiological data for these infections remains challenging [19]. In this systematic review, most cases were male, and the mean age was 42.08 years. Intriguingly, most cases were detected in European countries, while only 27.58% were observed in Asia and North or South America. Only 10.34% were noted in Oceania. The higher prevalence in Europe may be attributed to better surveillance systems or a relatively larger number of individuals with CF [36]. The low prevalence in Asian countries and the lack of reported cases in Africa reduces the possibility of an association between *Pandoraea* infection and poor living conditions. However, the limited number of published studies and the elevated misdiagnosis rates globally impede the establishment of solid epidemiological conclusions regarding *Pandoraea* infections.

The *Pandoraea* genus currently includes 28 formally recorded taxa, a figure that is constantly increasing [37,38]. It belongs to the Burkholderiaceae family and the Betaproteobacteria class [10,24]. Eleven species have been described, including *Pandoraea apista*, *P. norimbergensis*, *P. pulmonicola*, *P. sputorum*, and *P. pnomenusa* [39]. The pathogen is a Gram-negative, obligate aerobic, rod-shaped bacterium that shows oxidase variability, with around 64% being oxidase-positive. It is non-lactose-fermenting, motile thanks to a single polar flagellum, urea-positive, indole-negative, capable of growth at 42 °C on cetrimide and *Burkholderia cepacia*-selective media, but unable to grow on acetamide [3,9]. It has been retrieved from environmental sources, respiratory secretions, blood cultures, urine samples, and wounds [9,40]. *Pandoraea* infections were initially considered nosocomial and frequently isolated from patients on long-term ventilation [3]. *Pandoraea* is frequently misidentified as *Ralstonia* or *Burkholderia* species, as these two genera are strongly related to *Pandoraea* spp. phylogenetically and phenotypically [10]. The microorganism generally exhibits low invasive potential; however, cases with systemic infection and severe complications, such as sepsis, have occasionally been described [10,39]. 

Regarding potential predisposing risk factors, a history of cystic fibrosis was the most prevalent, according to the results of this systematic review. Cystic fibrosis constitutes a genetic disorder that provokes the accumulation of thick mucus in several organs, particularly the lungs; respiratory failure remains the principal cause of morbidity and mortality in CF patients [41]. This condition facilitates bacterial proliferation and severe respiratory infection. Non-fermenting Gram-negative bacteria are the most commonly isolated pathogens known to colonize the lungs [42]. However, several opportunistic pathogens invade the CF lungs through the years, often resulting in chronic infection with *P. aeruginosa* and, secondly, with *Burkholderia cepacian* complex (Bcc). Numerous opportunistic pathogens may also be isolated, including Enterobacteriaceae or *Stenotrophomonas maltophilia* [42,43]. *Pandoraea* spp. has recently been associated with CF as a newly emerging and multi-drug-resistant bacteria [19,44]. *Pandoraea*, and especially *P.pulmonicola* and *P. pnomenusa,* are capable of biofilm formation; this increases the pathogen’s virulence in patients with cystic fibrosis [45,46]. Moreover, most CF patients in this study had received antibiotics within the last three months. In most cases, the infection was polymicrobial, with *P. aeruginosa* being the most frequently isolated pathogen, potentially contributing to the severity of the clinical manifestations. To date, little data regarding *Pandoraea* species’ pathogenicity, mechanisms of drug resistance, and effect on CF lung are available; thus, more studies are required to elucidate their exact role in CF [19]. 

Another important risk factor observed in patients with *Pandoraea* infections was immunosuppression; active malignancy, either solid or hematologic, chemotherapy administration, autoimmune syndromes, and organ transplantation were observed in several cases. In most cases included in this review, antibiotics were repeatedly administered within the three months prior to infection. The repeated systematic use of antibiotics may cause microorganism selection and foster types of rare pathogen infections. Moreover, in patients with immunodeficiency, the asymptomatic colonization of the respiratory tract and other organs with *Pandoraea* is a high possibility; the development of systemic infection depends on the pathogens’ invasive potential [21]. Moreover, patients subjected to organ transplantation also receive intense immunosuppressive treatment; therefore, the proliferation of opportunistic pathogens is facilitated due to decreased immunity [47,48]. In this review, three patients underwent lung transplantation, two patients underwent liver transplantation, and one patient underwent allogenic stem cell transplantation due to hematologic malignancy. The use of CVC lines may also predispose to infection, especially in patients with bacteremia. CVCs provide entry points for pathogens into the bloodstream and enable bacterial attachment to the silicone tubing. Biofilm formation, observed in particular *Pandoraea* species, promotes bacterial proliferation and antimicrobial resistance [49]. In the present review, most patients with a CVC presented with bacteremia.

The identification of *Pandoraea* spp. remains challenging since most microbiology laboratories have limited access to advanced molecular methods, such as genetic testing. The detection of non-fermentative Gram-negative bacilli can be difficult, partly due to the exclusion of certain taxa from the databases of most commercial systems and partly because some species are non-reactive or exhibit slow growth [50]. Moreover, misidentification with *Ralstonia* or *Burkholderia* species is frequent, given their overlapping phenotypes [10]. High clinical and microbiological suspicions are essential for prompt identification. One key point in detecting the pathogen is the peculiar pattern of antimicrobial resistance to carbapenems, a distinctive characteristic of most *Pandoraea* species. Susceptibility tests have revealed significant multi-drug resistance as well as an exclusive pattern of carbapenem resistance: susceptibility to imipenem and resistance to meropenem [10,39]. Advanced microbiological procedures, such as 16s rRNA or matrix-assisted laser desorption/ionization time-of-flight mass spectrometry (MALDI-TOF MS), are generally necessary for more precise identification. In the present review, MALDI-TOF was the most common method used for pathogen identification, followed by 16s rRNA. In several cases, both methods were applied to successfully identify the pathogen. A study by Martina et al. demonstrated that a combination of 16s rRNA and mass spectrometry successfully detected *P. sputorum* infection that was initially misdiagnosed [12]. However, given these techniques’ high cost and low availability, successful diagnosis should also rely on specific pathogen biochemical or cultural features. In a study by Jorgensen et al., pulsed-field gel electrophoresis (PFGE) was used to conduct an epidemiological analysis of *P. apista* isolates [9]. PFGE is considered one of the optimal methods for epidemiological studies. However, it is laborious and time-consuming; as a result, considerable effort has been made to design techniques incorporating PCR [16]. Next-generation sequencing (NGS) technologies have advanced rapidly in recent years and offer the potential to detect pathogens by recognizing small amounts of DNA or RNA sequences at a reduced cost [51]. While NGS could serve as a diagnostic tool for the rapid and precise identification of *Pandoarea* spp., its specific role in diagnosing this pathogen has not yet been documented in the literature. Additional studies are needed to gather relevant data.

Until today, there have been no official or widely established antibiotic susceptibility breakpoints for *Pandoraea* spp., as no formal studies have evaluated the susceptibility profile of this species. As a result, the data in this review are based on case reports that include information on the pathogen’s antimicrobial susceptibility. Treatment should be carefully considered given the pathogen’s peculiar resistance patterns; *Pandoraea* spp. is considered multi-drug-resistant, and most species are resistant to β lactams and aminoglycosides [15]. A possible explanation for multi-drug resistance is the germ’s production of certain enzymes and efflux pumps. Specifically, oxacillinase-62 plays an important role against imipenem resistance [22]. Moreover, resistance may be attributed to chronic antibiotic administration in CF patients [3]. Since no definitive explanation has been provided regarding the pathogens’ resistance pattern, more thorough investigations regarding these resistance mechanisms and establishing specific criteria for interpreting sensitivity assays remain essential. 

In cases of systemic infection, aggressive antimicrobial treatment is indicated to prevent a fatal outcome [15]. According to the data in the present systematic review, carbapenems, especially imipenem, could be the optimal treatment for severe *Pandoraea* infection; in most cases, the pathogens were susceptible to imipenem. Cephalosporins and trimethoprim/sulfamethoxazole were also commonly administered amongst *Pandoraea*-infected patients, followed by aminoglycosides and quinolones. Antibiotic therapy should rely on actual in vitro susceptibility testing, considering the pathogen’s resistance pattern [39]. The treatment duration depends on the infection site and the symptoms’ severity; in this review, the duration ranged from 14 days to more than three months for severe complications, such as respiratory failure [9]. Surgical procedures were conducted in complicated cases, mainly consisting of CVC removal and replacement and lung transplantation, in cases with respiratory failure [15,16]. The mortality rate of *Pandoraea* infections was relatively high, especially in patients with cystic fibrosis or cases complicated by respiratory or multi-organ failure. High mortality rates may result from delayed diagnosis or misdiagnosis with other similar bacteria. The pathogen’s ability to produce serious infections, difficulty accessing suitable diagnostic tools, and unique resistance may also lead to elevated mortality rates [14,15].

This study is subject to certain limitations. The literature search may not have captured all the relevant studies on epidemiology and mortality, and the search strategy could have resulted in some studies being overlooked. Our analysis focused solely on case reports and case series, which rely on accurate record-keeping to maintain credibility. The exclusion of studies in languages other than English may potentially lead to a sample bias; however, the number of these articles was low. Additionally, in many studies, molecular identification methods such as 16s rRNA sequencing or MALDI-TOF MS were not utilized, raising the possibility of misidentification in some cases. Furthermore, some studies had incomplete data, limiting the scope of our statistical analysis to the available information. As a result, we could only present findings based on studies that provided complete data.

## 5. Conclusions

This study offers insights into the epidemiology, clinical features, microbiology, antimicrobial susceptibility, treatment, and outcomes of *Pandoraea* infections, highlighting key information on the pathogenic potential of this microorganism. *P. apista* was the most commonly identified species, while the respiratory system was the most frequently affected. Peculiar antimicrobial resistance patterns were observed, with resistance to meropenem and susceptibility to imipenem. Despite the lack of validated therapeutic guidelines, carbapenems were the most widely administered antibiotics. The site of infection affects patients’ outcomes, with its clinical severity closely linked to the patient’s immune status. Due to *Pandoraea’s* unique antimicrobial resistance pattern and capacity to induce severe infection, caution is advised when a pathogen cannot be definitively identified as either *B. cepacia* complex or *Ralstonia* species, and antibiotic treatment should be guided by actual in vitro susceptibility testing. Although the present review encompasses certain limitations, it may inspire further longitudinal investigations and controlled studies that will elucidate on *Pandoraea* infections and offer essential information on treatment strategies in the future. 

## Figures and Tables

**Table 1:** Characteristics of patients with *Pandoraea* species infection.

Characteristic	All Patients (*n* = 43) *	Survived (*n* = 30) *	Died (*n* = 13) *	*p*-Value
Age, years, median (IQR)	43 (22–61)	40 (21.8–61.3)	44 (28–61)	0.7788
Male gender, *n* (%)	25 (58.1)	17 (56.7)	8 (61.5)	1
Predisposing factors				
Post-surgery, *n* (%)	3 (7)	2 (6.7)	1 (7.7)	1
Post-cardiac surgery, *n* (%)	2 (4.7)	2 (6.7)	0 (0)	1
Cystic fibrosis, *n* (%)	17 (39.5)	14 (46.7)	3 (23.1)	0.1874
Immunosuppression, *n* (%)	8 (18.6)	4 (13.3)	4 (30.8)	0.2170
Active malignancy, *n* (%)	7 (16.3)	5 (16.7)	2 (15.4)	1
Central venous catheter, *n* (%)	5 (11.6)	4 (13.3)	1 (7.7)	1
Previous antimicrobial treatment, *n* (%)	15 (34.9)	10 (33.3)	5 (38.5)	0.7422
Bacteremia, *n* (%)	13 (30.2)	8 (26.7)	5 (38.5)	0.4854
Lower respiratory tract infection, *n* (%)	32 (74.4)	22 (73.3)	10 (76.9)	1
Polymicrobial infection, *n* (%)	23 (53.5)	19 (63.3)	4 (30.8)	0.0943
Clinical characteristics				
Fever, *n* (%)	14/32 (43.8)	7/23 (30.4)	7/9 (77.8)	0.0225
Sepsis, *n* (%)	13/36 (36.1)	5/25 (20)	8/11 (72.7)	0.0064
Treatment				
Cephalosporin, *n* (%)	14/39 (35.9)	12/27 (44.4)	2/12 (16.7)	0.1509
Carbapenem, *n* (%)	32/39 (82.1)	21/27 (77.8)	11/12 (91.7)	0.4026
Quinolone, *n* (%)	10/39 (25.6)	9/27 (33.3)	1/12 (8.3)	0.1312
Tetracyclines, *n* (%)	13/39 (33.3	10/27 (37)	3/12 (25)	0.7144
TMP-SMX, *n* (%)	14/39 (35.9)	12/27 (44.4)	2/12 (16.7)	0.1509
Outcomes				
Deaths due to infection, *n* (%)	9 (20.9)	NA	NA	NA
Deaths overall, *n* (%)	13 (30.2)	NA	NA	NA

IQR: interquartile range; NA: not applicable; TMP-SMX: trimethoprim-sulfamethoxazole; *: data are among the number of patients mentioned on top unless otherwise described.

**Table 2:** Antimicrobial resistance rates (TMP-SMX: Trimethoprim-Sulfamethoxazole).

Antimicrobial Agent	Number of Patients	Resistance (%)
Penicillin	6/6	100
Aztreonam	21/21	
Macrolides	7/7	
Chloramphenicol	7/7	
Aminoglycosides	27/28	96.42
Piperacillin	13/16	81.25
Quinolones	26/33	78.78
Cephalosporins	26/34	76.47
Piperacillin/Tazobactam	18/26	69.23
Aminopenicillins	13/23	56.52
Ticarcillin/Clavulanate	2/4	50
Tetracyclines	10/21	47.61
Carbapenems	6/35	17.14
TMP-SMX	3/34	8.82
Aminopenicillin + β-lactamase inhibitor	1/12	8.33

**Table 3:** Characteristics of the different types of *Pandoraea* species infections.

Characteristic *	Lower Respiratory Tract Infection (*n* = 32)	Bacteremia (*n* = 13)	Endocarditis (*n* = 2)	Pancreatitis (*n* = 2)	Upper Respiratory Tract Infection (*n* = 2)
Age, years, mean (IQR)	38.07	45.85	39.5	56	73.5
Male gender, *n* (%)	17 (53.12%)	10 (76.92%)	2 (100%)	1 (50%)	1 (50%)
Predisposing factors					
Post-surgery, *n* (%)	1 (3.12%)	3 (23.07%)	0	0	0
Post-cardiac surgery, *n* (%)	1 (3.12%)	1 (7.69%)	1 (50%)	0	0
Cystic fibrosis, *n* (%)	17 (53.12%)	2 (15.38%)	0	0	0
Immunosuppression, *n* (%)	6 (18.75%)	4 (30.76%)	1 (50%)	0	0
Active malignancy, *n* (%)	4 (12.5%)	3 (23.07%)	0	0	0
Central venous catheter, *n* (%)	2 (6.25%)	4 (30.76%)	1 (50%)	0	0
Previous antimicrobial treatment, *n* (%)	12 (37.5%)	4 (30.76%)	1 (50%)	2 (100%)	1 (50%)
Bacteremia, *n* (%)	4 (12.5%)	NA	2 (100%)	1 (50%)	1 (50%)
Lower respiratory tract infection, *n* (%)	NA	4 (30.76%)	0	0	2 (100%)
Polymicrobial infection, *n* (%)	20 (62.5%)	3 (23.07%)	0	1 (50%)	0
Clinical characteristics					
Fever, *n* (%)	8 (31.25%)	10 (76.92%)	2 (100%)	NR	1 (50%)
Sepsis, *n* (%)	8 (31.25%)	6 (46.15%)	1 (50%)	NR	1 (50%)
Treatment					
Cephalosporin, *n* (%)	11 (34.3%)	5 (38.46%)	0	0	0
Carbapenem, *n* (%)	26 (81.25%)	9 (69.23%)	1 (50%)	2 (100%)	2 (100%)
Quinolone, *n* (%)	6 (18.75%)	4 (30.76%)	1 (50%)	0	0
Tetracyclines, *n* (%)	10 (31.25%)	4 (30.76%)	1 (50%)	0	0
TMP-SMX, *n* (%)	11 (34.3%)	3 (23.07%)	1 (50%)	1 (50%)	0
Outcomes					
Deaths due to infection, n (%)	7 (21.87%)	4 (30.76%)	1 (50%)	0	0
Deaths overall, *n* (%)	10 (31.25%)	5 (38.46%)	1 (50%)	1 (50%)	1 (50%)

*: Data are among the number of patients mentioned on top unless otherwise described. IQR: interquarile range; NA: not applicable, NR: not reported; TMP-SMX: trimethoprim-sulfamethoxazole.

**Table 4:** Logistic regression of overall mortality of *Pandoraea* species infections.

Characteristic	Univariate Analysis *p*-Value	Multivariate Analysis *p*-Value	OR (95% CI)
Fever	0.0143	0.280	3.359 (0.372–30.297)
Sepsis	0.0017	0.153	4.658 (0.564–38.470)

CI: confidence interval; OR: odds ratio.

## Data Availability

The data presented in this study are available on request from the corresponding author.

## Supplementary Materials

The following supporting information can be downloaded at https://www.mdpi.com/article/10.3390/jcm13226905/s1: Table S1: Characteristics of included studies.
