# Supplementary material for: Pandoraea Infections in Humans—A Systematic Review
Source: J Clin Med. 2024 Nov 16;13(22):6905. doi: 10.3390/jcm13226905 (PMC11594697; doi:10.3390/jcm13226905)
Supplement: Supplementary file 1 [file jcm-13-06905-s001.zip › jcm-3306680-SI.pdf]

Table S1: Characteristics of included studies.

Author, Year	Number of Patients	Gender	Age (Years)	Site of Infection (%)	Treatment (%)	Mortality (%)
Moore et al., 2002	1	Male	17	Lower Respiratory (100)	Cephalosporine, Colistin, Quinolone, Aminoglycoside (100)	0 (0)
Stryjewski et al., 2003	1	Male	30	Lower Respiratory Infection, Bacteremia (100)	Carbapenem, Cephalosporin, Vancomycin (100)	1 (100)
Jorgensen et al., 2003	6	Female 6 (100%)	24	Lower Respiratory 6 (100)	Carbapenem, Cephalosporine, Macrolide, Aminoglycoside, Rifampicin, Tetracycline, TMP/SMX (100)	0 (0)
			21			
			21			
			22			
			20			
			22			
Johnson et al., 2004	1	Male	16	Lower Respiratory Infection, Bacteremia (100)	Carbapenem, Céphalosporine, Quinolone, Aminoglycoside, Tetracycline (100)	0 (0)
Atkinson et al., 2006	2	Female	30	Lower Respiratory 2 (100)	NR	0 (0)
		Male	36			
Pimentel et al., 2008	1	Male	32	Lower Respiratory (100)	Colistin, Aminoglycoside, Ticarcillin/ clavulanate (100)	0 (0)
Fernandez-Olmos et al., 2011	1	Female	30	Lower Respiratory (100)	Piperacillin/ Tazobactam, Carbapenem, Colistin, Quinolone, Macrolide, TMP/SMX (100)	0 (0)
Kokcha et al., 2013	1	Female	30	Lower Respiratory (100)	Carbapenem, Colistin, Tigecyclin, Aminoglycoside (100)	1 (100)
Pugès et al., 2015	1	Male	13	Lower Respiratory (100)	Carbapenem, TMP/ SMX	0 (0)
Ambrose et al., 2016	1	Male	26	Lower Respiratory (100)	Piperacillin/Tazobactam, Carbapenem, Vancomycin,	1 (100)

					Macrolide, Aminoglycoside, TMP/ SMX (100)	
Martina et al., 2017	1	Male	9	Lower Respiratory (100)	Carbapemen, Colistin, Quinolone, Aminoglycoside, TMP/ SMX (100)	0 (0)
Monzon et al., 2017	1	Male	79	Bacteremia (100)	NR	0 (0)
Lin et al., 2019	1	Male	44	Lower Respiratory (100)	Carbapenem, Vancomycin (100)	1 (100)
Xiao et al., 2019	1	Male	43	Bacteremia (100)	Céphalosporine, Carbapenem, Tazobactam (100)	0 (0)
Boutin et al., 2020	1	Female	48	Lower Respiratory (100)	Piperacillin/ Tazobacteam, Quinolone, Tigecycline (100)	0 (0)
Gawalkar et al., 2020	1	Male	42	Bacteremia, Endocarditis (100)	Piperacillin/ Tazobactam, Carbapenem, Vancomycin, Quinolone	1 (100)
Peyclit et al., 2021	1	Female	21	Lower Respiratory, Bacteremia (100)	Piperacillin/ Tazobactam, Carbapenem, Rifampicin, Tetracyclines (100)	1 (100)
Dlewati et al., 2021	1	Male	69	Lower Respiratory (100)	Cephalosporin, Carbapenem, Tetracycline (100)	1 (100)
Patil et al., 2021	1	Male	67	Osteomyelitis (100)	Quinolone (100)	0 (0)
Bodenoerfer et al., 2021	1	Male	37	Bacteremia, Endocarditis (100)	Piperacillin/ Tazobactam, Tigecycline, TMP/ SMX (100)	0 (0)
Singh et al., 2021	1	Male	72	Upper and Lower Respiratory, Bacteremia (100)	Aztreonam, Carbapenem, Macrolide, Hydroxychloroquine (100)	0 (0)
Ma et al., 2022	1	Female	46	Bacteremia, Skin (100)	Cephalosporine, Carbapenem, Quinolone, Tigecycline (100)	0 (0)
Itoh et al., 2022	1	Male	61	Bacteremia, Liver (100)	Piperacillin/ Tazobactam, Aminopenicillin, Quinolone, TMP/ SMX (100)	0 (0)
Cubidez-Diaz et al., 2022	1	Male	55	Lower Respiratory (100)	Aminopenicillin, Sulbactam, Cephalosporine, Carbapenem,	0 (0)

					Linezolid, Quinolone, TMP/ SMX (100)	
Oliveira et al., 2023	1	Female	51	Bacteremia (100)	Cephalosporine, Carbapenem, Avibactam, Daptomycin (100)	0 (0)
Zhang et al., 2023	1	Male	53	Bacteremia (100)	NR	1 (100)
Kim et al., 2023	1	Female	1.5	Lower Respiratory (100)	Antifungal agents	1 (100)
Kruis et al., 2023	10	Female 4 (40 %)<br><br>Male 6 (60%)	67<br>45<br>69<br>59<br>60<br>67<br>62<br>75<br>62<br>55	Lower Respiratory 8 (80%)<br>Pancreatitis 2 (20%)<br>Upper Respiratory 1 (10%)<br>Bacteremia 1 (10%)<br>Osteomyelitis 1 (10%)	Carbapenem 10 (100%)<br>TMP/ SMX 1 (10%)	4 (40%)
